# Variants in the human potassium channel gene (*KCNN3*) are associated with migraine in a high risk genetic isolate

**DOI:** 10.1007/s10194-011-0392-7

**Published:** 2011-10-22

**Authors:** Hannah C. Cox, Rod A. Lea, Claire Bellis, Melanie Carless, Tom Dyer, John Blangero, Lyn R. Griffiths

**Affiliations:** 1 Genomics Research Centre, Griffith Health Institute, Gold Coast Campus, Griffith University, Southport, QLD 4222 Australia; 2 Department of Genetics, Southwest Foundation for Biomedical Research, San Antonio, 78227 USA

**Keywords:** Migraine, Norfolk Island, Association, Population isolate, KCNN3

## Abstract

The calcium-activated potassium ion channel gene (KCNN3) is located in the vicinity of the familial hemiplegic migraine type 2 locus on chromosome 1q21.3. This gene is expressed in the central nervous system and plays a role in neural excitability. Previous association studies have provided some, although not conclusive, evidence for involvement of this gene in migraine susceptibility. To elucidate KCNN3 involvement in migraine, we performed gene-wide SNP genotyping in a high-risk genetic isolate from Norfolk Island, a population descended from a small number of eighteenth century Isle of Man ‘Bounty Mutineer’ and Tahitian founders. Phenotype information was available for 377 individuals who are related through the single, well-defined Norfolk pedigree (96 were affected: 64 MA, 32 MO). A total of 85 SNPs spanning the KCNN3 gene were genotyped in a sub-sample of 285 related individuals (76 affected), all core members of the extensive Norfolk Island ‘Bounty Mutineer’ genealogy. All genotyping was performed using the Illumina BeadArray platform. The analysis was performed using the statistical program SOLAR v4.0.6 assuming an additive model of allelic effect adjusted for the effects of age and sex. Haplotype analysis was undertaken using the program HAPLOVIEW v4.0. A total of four intronic SNPs in the KCNN3 gene displayed significant association (*P* < 0.05) with migraine. Two SNPs, rs73532286 and rs6426929, separated by approximately 0.1 kb, displayed complete LD (*r* ^2^ = 1.00, *D*′ = 1.00, *D*′ 95% CI = 0.96–1.00). In all cases, the minor allele led to a decrease in migraine risk (beta coefficient = 0.286–0.315), suggesting that common gene variants confer an increased risk of migraine in the Norfolk pedigree. This effect may be explained by founder effect in this genetic isolate. This study provides evidence for association of variants in the KCNN3 ion channel gene with migraine susceptibility in the Norfolk genetic isolate with the rarer allelic variants conferring a possible protective role. This the first comprehensive analysis of this potential candidate gene in migraine and also the first study that has utilised the unique Norfolk Island large pedigree isolate to implicate a specific migraine gene. Studies of additional variants in KCNN3 in the Norfolk pedigree are now required (e.g. polyglutamine variants) and further analyses in other population data sets are required to clarify the association of the KCNN3 gene and migraine risk in the general outbred population.

## Introduction

Migraine is a chronic and debilitating neurovascular disease characterised by recurrent attacks of severe headache that is usually accompanied by nausea, vomiting, photophobia and phonophobia. Clinical diagnosis is established by fulfilment of symptom-based criteria defined by the International Headache Society (IHS), which recognises two primary sub-types: migraine with aura (MA) and migraine without aura (MO) [1]. Migraine is highly prevalent in Western nations, afflicting some 12% of the adult population (males 5.6%, females 17.1%) [2]. The disorder has a strong genetic component, with population-based studies reporting heritability to vary from 0.34 to 0.57 [3]. However, heritability estimates as high as 0.96 for MA and 0.77 for MO have been reported in a Dutch population isolate [4]. Genetic liability is further supported by concordance rates in twins [5] and familial aggregation [6]. Non-genetic (environmental) factors are also thought to influence predisposition, with ethnic, geographic, lifestyle and socioeconomic factors shown to be associated with variable risk of migraine [7].

The molecular genetics of familial hemiplegic migraine (FHM), a rare mendelian variant of migraine may provide insight into the pathophysiology of more common forms of migraine. FHM is distinguished from general migraine by the presence of transient motor weakness accompanying typical aura symptoms. Mutations in the neuronal voltage-gated (P/Q-type) Ca^2+^ channel gene (*CACNA1A*; MIM601011) [8], the Na^+^/K^+^ ATPase gene (*ATP1A2*; MIM 182340) [9] and the neuronal voltage-gated Na^+^ channel gene (*SCN1A*; MIM182389) [10] have been identified in regions on chromosomes 19p13, 1q21-23 and 2q24, respectively. *CACNA1A* mutations produce gain-of-function of the Ca_V_2.1 channel and result in increased neurotransmitter release from cortical neurons [11]. Mutations in *ATP1A2* produce loss-of-function of the α2 Na^+^, K^+^-ATPase, whereas mutations in *SCN1A* accelerate recovery from fast inactivation of sodium-voltage channels [11]. The functional consequences of mutations in these genes support the involvement of ion and neurotransmitter disturbances in migraine pathophysiology [12–14]. The calcium-activated potassium channel gene (*KCNN3*; MIM602983) is in the vicinity of the FHM2 linked region on chromosome 1q21-23 region and proximal to a region linked to both FHM and typical migraine on chromosome 1q31 [15].

Calcium-activated potassium channels contribute to the after-hyperpolarisation in central neurons [16]. Small-conductance calcium-activated potassium channels such as *KCNN3* play a crucial role in determining neuronal firing rates via the after-hyperpolarisation that occurs during an action potential. *KCNN3* is widely expressed in the brain, is potassium selective, voltage independent, and has high sensitivity to increases in intracellular calcium levels that typically occur during an action potential [16, 17]. Variations in this gene may facilitate initiation of CSD and trigeminal sensitivity, thus making it a functionally plausible migraine candidate susceptibility gene.

Previous case–control association studies have investigated CAG repeats in exon 1 in Australian and German case–control migraine cohorts [18, 19]. Results from the Australian cohort (202 case; 221 controls) displayed a trend for migraineurs to have increased CAG repeat length compared to controls. The Mossner study (190 cases, 232 controls) also found evidence of an association with alleles of the second polyglutamine repeat in migraineurs [19]. These results suggest potential involvement of *KCNN3* in migraine susceptibility.

The present study sought to perform gene-wide SNP genotyping of the migraine candidate gene, *KCCN3*, in a unique multigenerational pedigree from the Norfolk ‘Mutiny on the Bounty’ population isolate. These individuals are the living descendents of nine Isle of Man, ‘Bounty’ Mutineers and six Tahitian women, who colonised the uninhabited Pitcairn Island in 1790 after the infamous Mutiny aboard the HMAS Bounty. This initial Pitcairn Island colony experienced population bottlenecks and was characterised by minimal migration and consanguinity. The small community expanded in severe geographic and genetic isolation until 1856, when they were relocated to the then uninhabited Norfolk Island, located almost 1,500 km west of Brisbane, Australia in the South Pacific Ocean. The Norfolk pedigree is a novel resource to evaluate *KCNN3* gene involvement in migraine susceptibility.

## Methods

### Subjects

The study was approved by the Griffith University Human Research Ethics Committee prior to commencement. Prior to participation signed, informed consent was obtained from all participants. Data collection procedures have been described in detail elsewhere [20]. Phenotypic data and biological specimens (venous blood) were obtained from 600 subjects (261 males, 339 females) with a mean age of 50.8 years (standard deviation of 16.4 years). DNA was isolated using a standard salting-out procedure [21]. Phenotypic data were obtained via a comprehensive medical questionnaire that surveyed migraine family history, symptoms, triggers, and medication use. Migraine was diagnosed in accordance with current IHS guidelines by interviews using a validated migraine questionnaire and followed up by qualified migraine diagnosticians [1]. In total, 600 subjects participated in the study, of whom 154 (25.7%) were positive for migraine (MA and/or MO) of the total cohort. The rate of migraine in the Norfolk population is substantially higher than the estimated 12% affection rate in general, out-bred, Caucasian populations [2]. This finding reflects the unique population sub-structure, a highly complex, 11-generation pedigree originating from a limited number of founding individuals. At the time of the study, approximately 80% of permanent residents on the island were of ‘*Bounty*’ mutineer lineage [22]. This population is characterised by founder effect, geographical and cultural isolation, high levels of consanguineous unions during early population expansion, population bottlenecks, admixture and homogenous environment [20]. These factors may impact significantly on the frequency of particular traits or diseases as well as genetic drift within a population, as demonstrated by the high levels of founder mutations in the Dutch population [23].

Genealogical data were obtained from multiple sources, including questionnaire, municipal and historical records. Historically, Pitcairn Island was settled by 9 Isle of man ‘Bounty’ mutineers, 12 Tahitian women and 6 Tahitian men in 1790. Pedigree reconstruction and validation indicate that the present population are the descendents of the original 9 Isle of Man ‘Bounty mutineers’ founders, 6 Tahitian female founders and 2 additional Caucasian sailors who joined the small colony during the early nineteenth century [24]. Including recent married-in individuals, a total of 377 participants are related through the Norfolk Island ‘Mutiny on the Bounty’ pedigree. In total, 96 (25.5%) of the 377 pedigree members were migraine positive. To facilitate analysis, the original pedigree (*N* = 6,537) was split (*N* = 1,078) using a peeling algorithm in the pedigree database management system, PEDSYS (Southwest Foundation for Biomedical Research, San Antonio, TX, USA) [25]. This 1,078-member pedigree has been previously employed in genome-wide screens of cardiovascular risk traits [26].

### Genotyping

Genotyping was performed in a sub-sample of 285 related individuals (135 males, 150 females) selected from the core 377-member pedigree. These individuals possessed high inheritance information and were extremely informative for pedigree-based analysis, facilitating cost-effective genotyping. Of these related individuals, 76 are migraine positive (22 males, 54 females). DNA samples were genotyped according to the manufacturer’s instructions on an Illumina Infinium DNA analysis BeadChip requiring 200 ng of DNA per sample. A total of 85 SNPs were genotyped across the *KCNN3* gene. Samples were scanned on the Illumina BeadArray 500GX Reader. Raw data were obtained using Illumina BeadScan image data acquisition software (version 2.3.0.13) and analysed using Illumina BeadStudio software (version 1.5.0.34). Individuals with a call rate below 95% and SNPs with a call rate below 99%, deviating from Hardy–Weinberg equilibrium or with a minor allele frequency of less than 1% were excluded from the analysis. The Pedigree RElationship Statistical Test (PREST) was used to verify the pedigree structure and detect relationship misspecification. Genotypic data were analysed for discrepancies using the PEDSYS program INFER and Simwalk2. Discrepant genotypes were blanked prior to analysis.

### Statistical analysis

All statistical analyses on related individuals were conducted using variance component-based methodology implemented in the Sequential Oligonucleotide Linkage Analysis Routines (SOLAR) version 4.0.6 software package (Southwest Foundation for Biomedical Research, San Antonio, TX, USA) [27]. Gene-wide association testing was performed using measured genotype analysis, embedded in a variance component-based linkage model [28]. The analysis performed assumed an additive model of allelic effect, where SNP genotypes AA, AB and BB were coded as −1, 0 and 1, respectively, and used as a linear predictor of the phenotype. The analysis was adjusted for the covariate effects of age, age-squared, sex and their interactions to allow for differential symptom prevalence in males and females and adjust for variable age of onset. A total of 85 SNPs across the *KCCN3* gene were available for the analysis. The local type I error, *α* = 5.8 × 10^−4^ was calculated by Bonferroni adjustment. SNP results were annotated using the Whole Genome Association Study Viewer (WGAViewer) program (http://people.genome.duke.edu/~dg48/WGAViewer/) and NCBI Build 37.1 [29]. Linkage disequilibrium was assessed using the program Haploview [30]. Due to the presence of a breeding loop in the trimmed pedigree file, the pedigree was further split to facilitate Haploview analysis within a pedigree framework, using the partitioning program Jenti [31].

## Results

This study undertook pedigree-based association testing to determine involvement of the KCNN3 gene in susceptibility to familial migraine in a unique isolated population from Norfolk Island. Of the 85 SNPs within the *KCNN3* gene on chromosome 1, four displayed nominal level association with migraine. Two of the top four SNPs were determined to be in complete LD (*r* ^2^ = 1.0, *D*′ = 1.00, *D*′ 95% CI = 0.96–1.00). Beta coefficients and corresponding *P* values for the four significant SNPs are detailed in Table 1. The probit regression beta coefficient is a measure of risk. A negative beta indicates that the minor allele increases migraine risk, a positive beta indicates a decreased risk. The minor allele for the four significant SNPS conferred a decreased risk of migraine in the Norfolk pedigree. This suggests that the common gene variants in *KCNN3* may confer an increased risk of migraine in the Norfolk pedigree.

**Table 1:** Summary of *KCCN3* SNPS displaying gene-wide significance

dbSNP rs no.	NCBI build 37.1 position (bp)	Function	Beta	*P* value
rs4845663	154692088	Intronic	0.286	0.023
rs7532286^a^	154750816	Intronic	0.283	0.024
rs6426929^a^	154750989	Intronic	0.283	0.024
rs1218551	154801173	Intronic	0.315	0.038

^a^SNPs displaying complete LD (*r* ^2^ = 1.0, *D*′ = 1.00, *D*′ 95% CI = 0.96–1.00)

The Norfolk population isolate is unique due to the presence of population admixture during the founding event. The degree of Polynesian and Caucasian admixture in the Norfolk pedigree has been previously estimated using the ancestry coefficient (*Q*) [24]. Polynesian and Caucasian ancestries were estimated as 12 and 88%, respectively. Due to this unique population structure we compared the minor allele frequencies of our top four SNPS with those reported in Phase III data from the Human HapMap populations (http://hapmap.ncbi.nlm.nih.gov/). Results are detailed in Table 2. Allelic frequencies in the Norfolk pedigree are similar to the CEU population.

**Table 2:** SNP minor allele frequencies in Norfolk Islanders compared to other populations

dbSNP rs no.	Minor/major allele	Minor allele frequency (MAF)				
		NI	CEU (C)	CHB (H)	JPT (J)	YRI (Y)
rs4845663	C/T	0.478	0.411	0.331	0.217	0.935
rs7532286	A/C	0.371	0.420	0.281	0.323	0.701
rs6426929	A/G	0.371	0.420	0.283	0.323	0.476
rs1218551	G/A	0.265	0.341	0.036	0.084	0.153

*CEU* (*C*) Utah residents with Northern and Western European ancestry from the CEPH collection, *CHB* (*H*) Han Chinese in Beijing, China, *JPT* (*J*) Japanese in Tokyo, Japan, *NI* Norfolk Islanders, *YRI* (*Y*) Yoruba in Ibadan, Nigeria

## Discussion

*KCNN3* is widely expressed throughout the brain and CNS, but is predominant in the hypothalamus, particularly in neurosecretory neurons (gonadotropin releasing hormone, dopamine and vasopressin neurons) [32]. There is also an evidence to suggest that oestrogen levels may influence *KCNN3* expression and possibly neuronal excitability [32]. Considering the role of *KCCN3* in neural firing via ion transport, as well as its relation to dopaminergic and oestrogen pathways, it can be considered a potential modifying gene in migraine susceptibility. The influence of circulating oestrogen levels on *KNCC3* expression may explain the sex-specific variation in migraine incidence and prevalence observed across age categories [33]. Risk variants and haplotypes in dopamine metabolism genes, *DBH* [34], *DDC* [35], *MAOA* [35], and *SLC6A3* [36], are reported to influence migraine risk, as are variants in the oestrogen receptor gene, *ESR1* [37, 38]. Whilst conflicting results are reported in different populations [39, 40], overall evidence suggests that genes involved in neurotransmitter pathways, particularly ion channel function and transport, are good migraine candidates.

Association between *KCNN3* polyglutamine repeat expansion length and migraine susceptibility has been demonstrated in two independent studies [18, 19]. To determine whether variants in the *KCNN3* gene are associated with migraine risk, we utilised a small genetic isolate from Norfolk Island and performed a comprehensive 85 SNP analysis of this gene. The multigenerational ‘Mutiny on the Bounty’ pedigree is a unique resource for genetic studies. Pedigree characteristics include 17 founding individuals, admixture, as well as cultural and geographical isolation. At the time of recruitment, the Island’s population of permanent residents under the Norfolk Island Immigration Act 1980 totalled 1,574 individuals [22]. Migraine was not analysed as a single phenotype as our *hypothesis* was that a common gene(s) basis is involved in migraine generally in accordance with Nyholt et al. [41]. Furthermore, a small sample size made subgroup analysis statistically impractical. A migraine prevalence (MA and/or MO) of 25.7% was observed in a population sample of 600 individuals. The most significantly associated SNPs (*P* < 0.005) in this pedigree were rs4845663, rs7532286, rs6426929 and rs1218551, with the minor allele in each case conferring protection against migraine risk.

A recent study of ion transport genes and migraine susceptibility provided evidence for a potential role of the potassium, voltage-gated, ion channel, *KCNE2* gene on chromosome 21q22.11 [42]. Of particular relevance to our current study were results of *KCCN3* gene screening in the Finnish case–control cohort [42]. Multiple SNPs within the KCCN3 gene and in the vicinity of the markers included in this study produced nominal level allelic association with migraine. Similar to findings in the Norfolk pedigree, a protective role was implicated for a *KCCN3* allelic variant and MA susceptibility (rs11810841; *P* = 0.0081; OR = 0.8232). Although different SNPs were implicated by Nyholt et al. [42] in *KCNN3*, when these results are considered with the findings of Curtain et al. [18] and Mössner et al. [19], they add substantial weight to our new findings in Norfolk Islanders. Overall results in the Norfolk pedigree, and Finnish, German and Australian populations suggest that common variants of small effect size in the *KCCN3* gene may influence migraine susceptibility. Additional analyses are required to determine if variants in *KCNN3* are associated with migraine risk. However, it is difficult to conduct follow-up studies of the Norfolk pedigree because of the uniqueness of our cohort, which includes characteristics such as founder effect, geographical and cultural isolation, high levels of consanguineous unions during early population expansion, population bottlenecks, admixture and homogeneous environment. Such unique population qualities can prohibit follow-up studies [43].

The Nyholt et al. [42] study screened 155 ion transport genes [42]. Variants within 12 of these genes displayed nominal associations. Evidence for epistatic interaction between potassium and calcium channel genes, *KCNB2* and *CACNB2*, was also detected. Based on the findings of this study, nominal associations found within KCNN3 may be further explored by screening ion channel genes, particularly potassium channels, and/or genes involved in oestrogen and dopamine metabolism and assessing potential gene–gene interactions.

Recently, a frameshift mutation in the potassium ion channel gene, *KCNK18*, was found to segregate with autosomal dominant MA in a multigenerational pedigree [44]. High expression levels of *KCNK18* were detected in the trigeminal ganglion, which has long been implicated as the initiation point for neural, vascular and inflammatory events that underlie typical migraine symptoms. Although disruption of ion channels and transporters are known to cause FHM, the Lafreniere study is the first to confirm ion channel disruption in a common migraine subtype, MA. Although the phenotype was a highly penetrant, autosomal dominant form of MA, results provide compelling evidence that variants in other ion channel genes, in particular, potassium channels such as *KCNN3*, may influence more common, complex forms of migraine. Findings in the Norfolk pedigree are consistent with the proposed connection between ion channel disruption and this complex disorder.

## Conclusion

This study provides evidence for association of multiple variants in the *KCNN3* ion channel gene with migraine susceptibility in a large multigenerational pedigree from the unique Norfolk Island genetic isolate. This the first comprehensive analysis of this potential candidate gene in migraine and also the first study that has utilised the unique Norfolk Island large pedigree isolate to implicate a specific migraine gene. Studies of additional variants in *KCNN3* in the Norfolk pedigree are now warranted, including the previously studied polyglutamine tracts to determine if these CAG repeats also show association with migraine in the Norfolk isolate.
